# Application of Potassium Humate and Salicylic Acid to Mitigate Salinity Stress of Common Bean

**DOI:** 10.3390/life13020448

**Published:** 2023-02-05

**Authors:** Hossam S. El-Beltagi, Hala Hazam Al-Otaibi, Aditya Parmar, Khaled M. A. Ramadan, Allan Klynger da Silva Lobato, Mohamed M. El-Mogy

**Affiliations:** 1 Agricultural Biotechnology Department, College of Agriculture and Food Sciences, King Faisal University, Al-Ahsa 31982, Saudi Arabia; 2 Biochemistry Department, Faculty of Agriculture, Cairo University, Gamma Street, Giza 12613, Egypt; 3 Food and Nutrition Science Department, Agricultural Science and Food, King Faisal University, Al-Ahsa 31982, Saudi Arabia; 4 Natural Resources Institute, University of Greenwich, Central Avenue, Chatham Maritime, Kent ME4 4TB, UK; 5 Central Laboratories, Department of Chemistry, King Faisal University, Al-Ahsa 31982, Saudi Arabia; 6 Department of Biochemistry, Faculty of Agriculture, Ain Shams University, Cairo 11241, Egypt; 7 Nucleo de Pesquisa Vegetal Basica e Aplicada, Universidade Federal Rural da Amazonia, Paragominas 68627-450, Para, Brazil; 8 Vegetable Crops Department, Faculty of Agriculture, Cairo University, Giza 12613, Egypt

**Keywords:** abiotic stress, antioxidant enzymes, humic acid, *Phaseolus vulgaris*, sodium chloride

## Abstract

In the current study, we investigated the effect of potassium humate (Kh) and salicylic acid (SA) in mitigating the salinity stress of common bean plants. Common bean seedlings were treated with 0.2 g/L SA as a foliar application and 0.3 g/L Kh as a soil application individually or in combination. After 7 days of germination, plants were treated with 50 mM NaCl and normal water as a control. Our results indicate that salt treatment reduced the plant growth (fresh and dry shoots and roots), leaf pigments (total chlorophyll and carotenoids), ascorbic acid (AA), glutathione (GSH), and potassium (K) contents. On the contrary, proline content; sodium (Na); hydrogen peroxide (H_2_O_2_); superoxide anion (O_2_^•−^); and antioxidant enzymes, including catalase (CAT), peroxidase (POX), and superoxide dismutase (SOD), were increased by saline stress. However, applying either individual Kh and SA or their combination stimulated seedling growth under salinity stress by increasing growth parameters, leaf pigment contents, AA, GSH, proline content, K content, and antioxidant enzymes compared with the control. Additionally, Na content, H_2_O_2_, and O_2_^•−^ were reduced by all applications. The application of the Kh (0.3 g/L) + SA (0.2 g/L) combination was more effective than using the individual compounds. In conclusion, applications of Kh + SA can mitigate salt stress and improve the seedling growth of common bean.

## 1. Introduction

Water and soil salinity have been among the main problems in the agricultural sector in recent decades. Most economically essential crops are classified as moderate or severely sensitive to salinity. These crops have been reported to lose about 20–50% of their production due to exposure to salinity stress [1]. More than 33% of agricultural lands are affected by salinity. Current estimates suggest that salinization is increasing by 10% annually due to factors such as irrigation with saline water, climate change, high temperatures, and high soil evaporation, as well as poor agricultural practices [2]. It is estimated that by 2050, about 50% of cultivated lands will be affected by salinity [3].

Crops grown under salinity stress suffer from osmotic stress and a lack of nutrient supply [4]. These factors cause severe damage to plants, such as reductions in seed germination, photosynthesis processes, growth, yield, and fruit quality [5,6,7]. Additionally, several physiological processes in plants, such as respiration, protein synthesis, lipid metabolism, enzyme and hormone activities, and gene expression, are affected by salinity [8,9]. The effect of salinity on plants occurs first during an early stage of growth known as the osmotic phase and then during a late stage of growth known as the ionic phase [10]. Plant response to salinity depends on the salinity level, growth stage, and exposure duration.

The common bean (*Phaseolus vulgaris* L.) is a notable crops belonging to the legume family that is consumed either as fresh pods (also known as green beans, French beans, string beans, or snap beans) or dry seeds. Common beans are considered one of the most nutritious vegetable crops due to the high availability of protein and micronutrients [11]. Globally in 2020, the production of common beans was about 23 million tonnes harvested from about 16 million hectares (FAOSTAT 2020, https://www.fao.org/faostat/en/, accessed on 1 September 2022). The top producers of green beans are China, Indonesia, Turkey, India, Thailand, and Egypt. 

One of the critical aspects of common bean cultivation is its salinity sensitivity [12]. It is well known that the growth and production of common bean plants are reduced when salinity levels exceed 1 dS m^−1^ [12]. Several previous studies have reported the adverse effect of salinity on the growth and production of common bean [13], as well as other legumes, such as soybean, mungbean, and fava bean [14,15,16]. Under saline conditions, Na^+^ and Cl^−^ are accumulated in plant cells, which causes toxicity for plants and reduces the uptake of most elements, such as N, P, K, Ca, and Mg [17]. Some of the strategies to reduce the harmful effects of salinity are the application of fertilizer with organic matter, including compost and biochar [18], foliar application with nutrients [19], the use of plant growth-promoting rhizobacteria [20], and breeding for resistance cultivars [21]. However, some previous applications have had limited effects. Therefore, more than one application may support mitigation of abiotic stresses crops sensitive to salinity, such as common beans. 

Potassium humate (Kh) is a salt derived from humic acid (HA) that is used to improve plant growth and production via soil [22] and foliar applications [23]. The role of HA in stimulating plant growth and increasing yield is attributed to its role in facilitating the transfer of elements from the soil into the plant by increasing the permeability of the cell membrane [24]. Additionally, it has been reported that HA improves plant growth and photosynthesis processes under abiotic stresses and increases antioxidant enzyme activity [25]. Additionally, potassium (K) is classified as a macro element essential for most physiological processes inside plants [26]. K plays a positive role in alleviating stresses such as salinity and drought [27]. Previous studies indicate the role of HA in mitigating salinity stress in crops such as sorghum [28]. A limited number of studies have been conducted on the effect of exogenous Kh application on beans, such as that by Taha and Osman [29], who found that Kh improves bean growth under saline stress. 

Salicylic acid (SA) is classified as a natural plant hormone and one of the phenolic compounds that affect several physiological processes, such as photosynthesis, respiration, growth, and production of plants [30]. Most reports indicate the positive role of foliar application with SA in resistance to salinity in different crops, such as strawberries [31], cabbage [32], and cowpea [33]. Previous studies reported the role of exogenous SA application in mitigating biotic and abiotic stresses [34,35].

The combination of SA as a foliar application and Kh as a soil application to mitigate abiotic stresses is rarely reported in the literature. For example, Shalaby et al. [36] found that foliar application of SA + Kh enhanced plant growth, leaf pigments, and the activities of the antioxidant enzymes of marigold plants. Additionally, foliar application of SA + Kh enhanced the growth, photosynthetic pigments, and K accumulation of maize seedlings compared to control plants [37].

To the best of our knowledge, the combined application of SA + Kh to mitigate salinity stress has not been evaluated to date in common bean plants. Our hypothesis in this study is that using SA + Kh could mitigate the salinity stress of common bean plants better than the individual application of either SA or Kh. To evaluate our hypothesis, SA as foliar application and Kh as soil application were applied to common bean plants, and their effects on the plant growth, nutrient uptake, and changes in non-enzymatic antioxidant and antioxidant enzymes were evaluated. The accumulation of reactive oxygen species and hormone formations was also tested.

## 2. Materials and Methods

### 2.1. Plant Materials and Treatments

**Figure 1:**
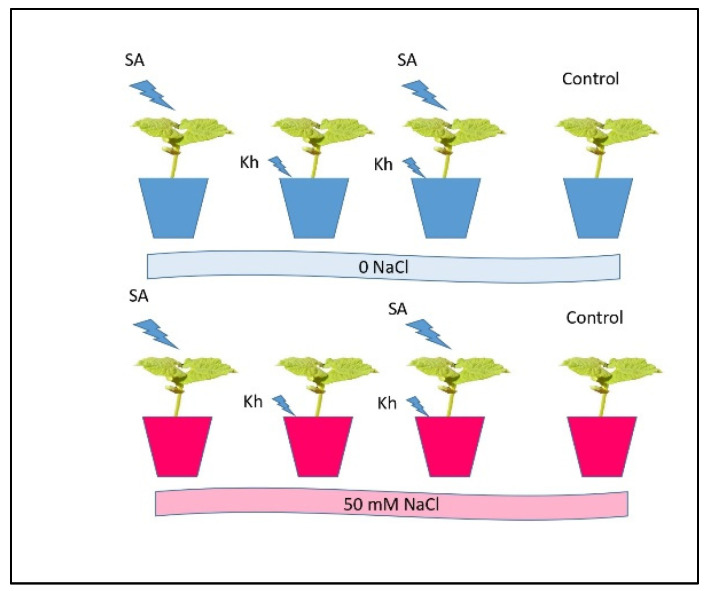
Scheme of the experimental treatments.

Pot experiments were performed in a modified growth chamber at a temperature of 24–28 °C of temperature, 65% relative humidity, and 3500 lx light intensity. Seeds of common bean (cv. Bronco) were used in this experiment. The seeds were sterilized with sodium hypochlorite (1%) for 2 min to avoid seed contamination before seed furrow. The seeds were washed twice with distilled water, then dried. The experiment consisted of 8 treatments (Figure 1) with 5 replicates as follows:

1. Soil application of K-humate treated with 0 NaCl (Kh + 0 NaCl);
2. Soil application of K-humate treated with 50 mM NaCl (Kh + 50 NaCl);
3. Foliar application of salicylic acid treated with 0 NaCl (SA + 0 NaCl);
4. Foliar application of salicylic acid treated with 50 mM NaCl (SA + 50 NaCl);
5. Soil application of K-humate + foliar application of salicylic acid + 0 NaCl (Kh + SA + 0 NaCl);
6. Soil application of K-humate + foliar application of salicylic acid + 50 mM NaCl (Kh + SA + 50 NaCl);
7. Foliar application of water treated with 50 mM NaCl (Cont. + 50 NaCl);
8. Control (without K-humate, salicylic acid, or NaCl) (Cont.).

The concentration of Kh was 0.3 g/L, and that of SA was 0.2 g/L according to previous work [38]. Potassium humate contains 65% humic acid and 15% potassium. 

The seeds were sown in plastic pots (15 × 15 cm) filled with acid-washed sand and arranged in a complete randomized design with 5 replicates. After 7 days of complete germination (14 days from seed planting), the desired salt concentration (200 mL) was added daily. Foliar spraying with SA (about 10 mL) was performed using a handgun sprayer on all shoots until the solution began to drip. The soil application of Kh was performed by adding the desired concentration (200 mL) into the growth media. Half-strength Hoagland’s nutrient solution was used to irrigate seedlings with saline treatment every two days. The foliar application of SA and soil application of Kh were performed 4 times 7, 14, 21, and 28 days after complete germination individually or together. After 33 days of complete germination, the plants were harvested to determine the physiological and chemical parameters.

### 2.2. Plant Growth and Leaf Pigments

We used a digital balance to measure the fresh weight of the shoots and the roots. Plants were dried in a forced-air drying oven to measure the dry weight of shoots and roots (75 °C) until a consistent weight. For chlorophyll and carotenoid, the method described by Lichtenthaler and Wellburn [39] was followed. In brief, 1 g of fresh samples was extracted in 10 mL acetone (80%) for 1 h at 5 °C. Then, the samples were centrifuged for 15 min at 3000× *g*. The absorption was measured by a spectrophotometer (model UV-2401 PC, International Equipment Trading LTD. (IET), Milano, Italia) at three wavelengths (470, 647, and 663 nm) against the blank. Chlorophyll content and carotenoids are represented in the results as mg g^−1^ FW. 

### 2.3. Proline Content

The method of Bates et al. [40] was used to determine proline content. Briefly, leaf samples (0.1 g) were extracted in sulfosalicylic acid (3% 10 mL). Then, filter paper (Whatman one) was used to filter the samples. Subsequently, ninhydrin and glacial acetic acid (100% 2 mL) were mixed with 2 mL of the filtrated solution. The samples were boiled for one hour in a water bath at 100 °C. The samples were kept in ice water for 15 min, 4 mL of toluene was added, and the mixture was added and stirred for 15 to 20 s in a test tube. A spectrophotometer measured the absorbance at 520 nm. A standard curve was used to calculate the proline content, and the results are represented as µg g^−1^ FW.

### 2.4. Hydrogen Peroxide (H_2_O_2_) and Superoxide Anion (O_2_^•−^) 

The content of H_2_O_2_ in fresh leaves was measured according to the method described by Junglee et al. [41]. In brief, 0.1 g of fresh sample was homogenised with 1 mL of extraction mixture (0.1% TCA, 1 M KI, and 10 mM potassium phosphate buffer) for 10 min in an ice bath. The results are expressed as mmol g^−1^ FW. To determine O_2_^•−^, the method of Yang et al. [42] was followed. In brief, a fresh sample (0.2 g) of bean leaves was mixed with 3 mL of phosphate buffer (50 mM; pH 7.8). Polyvinylpyrrolidone (PVP 1% *w*/*v*) was mixed with the reagent and centrifuged for 20 min under cooling (4 °C) at 10,000 rpm. Finally, the optical density was measured at 530 nm. The results are expressed as mmol g^−1^ FW.

### 2.5. Determination of Antioxidant Enzymes

Anitoxidant enzymes in leaf samples were determined using the method proposed by Grace and Logan [43]. Proteins were extracted by homogenising 0.1 g of fresh samples in a buffer of potassium phosphate (pH 7.0), which contains 0.1 mM EDTA, 4% PVP, and 2% glycerol. Then, the samples were centrifuged at 15,000× *g* under cooling (4 °C) for 40 min. The supernatant was used as a crude extract. The total soluble protein was also determined in the supernatant to calculate the specific activity of different enzymes according to Bradford [44]. Enzyme activities of POX and CAT were measured in the supernatants.

To measure the activity of peroxidase (POX) (EC: 1.11.1.7), the method of Lagrimini [45] was used. In brief, 100 µL of crude extract was mixed with 2.9 mL of the reaction solution containing 100 mM guaiacol, 0.1 mM EDTA, 50 mM phosphate buffer, and 30 mM H_2_O_2_. The tetra-guaiacol was measured at 465 nm, and the POX activity result is presented as µmol mg^−1^ protein min^−1^. The activity of catalase (CAT) (EC: 1.11.1.6) was measured according to Aebi [46]. The ability of the enzyme extract to decompose H_2_O_2_ at room temperature was used to measure CAT activity by observing the drop in absorbance at 240 nm. The reaction mixture consisted of potassium phosphate (100 Mm) buffer (pH 7) and 25 mM H_2_O_2_. A volume of 100 µL of crude extract was added to 3 mL to begin the reaction. The results are expressed as µmol mg^−1^ protein min^−1^. The method of Dhindsa et al. [47] was used to determine the superoxide dismutase (SOD) activity. This method depends on the ability of one unit of SOD to inhibit 50% of nitro blue tetrazolium photoreduction at 560 nm. The results are expressed in mg^−1^ protein min^−1^.

### 2.6. Determination of Sodium (Na^+^) and Potassium (K^+^) in Leaves

The amounts of sodium (Na^+^) and potassium (K^+^) in bean leaves were measured using the flame photometric method (Jenway, Leicestershire, UK) as described previously [4]. In brief, the fresh samples were washed with distilled water and kept in a forced-air oven until constant weight at 70 °C. The dried samples were ground to obtain powder. Then, 0.2 g of sample was digested using a mixture of perchloric acids and sulphuric acid. The results are presented as mg g^−1^ DW.

### 2.7. Determination of Ascorbic Acid (AA) and Glutathione (GSH)

The previously described by Mukherjee and Choudhuri [48] was followed to determine the ascorbic acid (AA) content in the common bean leaves. Briefly, samples (0.1 g) were extracted in 6% (*w*/*v*) trichloroacetic acid. Then, 2 mL of dinitrophenyl hydrazine (2%; *w*/*v*) was mixed with 4 mL of the extract, and 1 drop of thiourea in 70% (*v*/*v*) ethanol was added. The mixtures were then boiled for 15 min in a water bath. After cooling, 5 mL of H_2_SO_4_ (80%; *v*/*v*) was added, and samples were read at 530 nm. The results are expressed as mg/100 g FW. The method of Griffth [49] was used to determine the concentration of GSH. The concentration of GSH was calculated from a standard curve, and the results are expressed as nmol GSH g^−1^ FW.

### 2.8. Statistical Analysis

The results were statistically analysed using a one-way analysis of variance under a complete randomized design. The means were compared by Duncan’s multiple range test at *p* ≤ 0.05. SPSS (V.21) computer software was used for statistical analysis. The heatmap figure was created using ClustVis online software. Principal component analysis (PCA) was performed using Statistica 7 software (TIBCO Software, Palo Alto, CA, USA).

## 3. Results

### 3.1. Effect of Kh and SA on Growth

**Figure 2:**
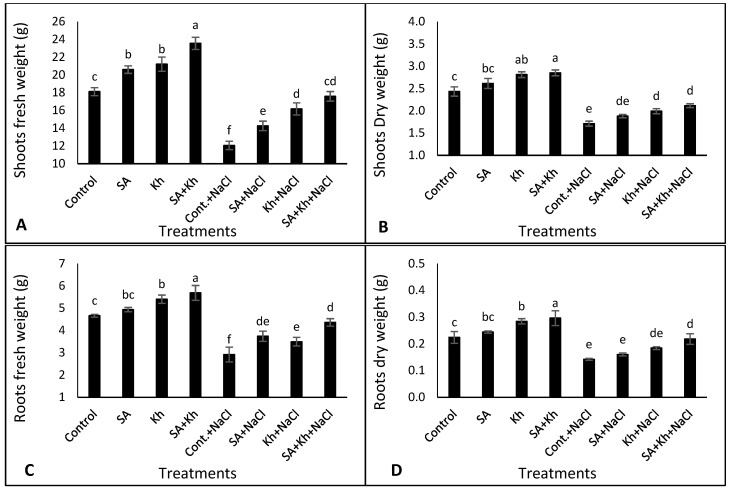
Effect of potassium humate (Kh), salicylic acid (SA), and their combination on (**A**) shoot fresh weight, (**B**) shoot dry weight, (**C**) root fresh weight, and (**D**) root dry weight of common bean plants irrigated with 0 and 50 mM NaCl. Different letters indicate significant differences according to Duncan’s test (*p* < 0.05). Data are reported as means ± SE; *n* = 5.

As expected, salinity stress (50 mM NaCl) reduced the growth of common bean plants (Figure 2A–D). Under non-saline and saline conditions, all treatments increased the fresh weight of shoots compared to control plants (Figure 2A). The highest values of shoots were recorded under the Kh + SA treatment. Furthermore, there was no significant difference in the fresh weight of shoots between the Kh + SA treatment under saline conditions and the control plants under non-saline conditions. Similarly, under non-saline conditions, Kh and Kh + SA applications showed higher shoot dry weight than the control plants (Figure 2B). Under the saline condition, both Kh and Kh + SA applications improved the shoots’ dry weight by 16.35 % and 23.59 %, respectively, compared with the control plants. 

As shown in Figure 2C, the root fresh weight was increased by Kh and Kh + SA treatments compared with control plants under non-saline conditions. However, under 50 mM NaCl stress, all treatments significantly increased the root fresh weight by 28.10%, 19.73%, and 49.41%, respectively, compared with the control. Furthermore, the Kh + SA treatment under saline conditions resulted in the highest values. As shown in Figure 2D, Kh and Kh + SA applications increased the dry weight of roots compared to the control treatment under non-saline conditions. Moreover, under saline conditions, Kh + SA treatment resulted in the highest values of root dry weight. 

### 3.2. Effect of Kh and SA on Leaf Pigments, O_2_^•−^, and H_2_O_2_

**Figure 3:**
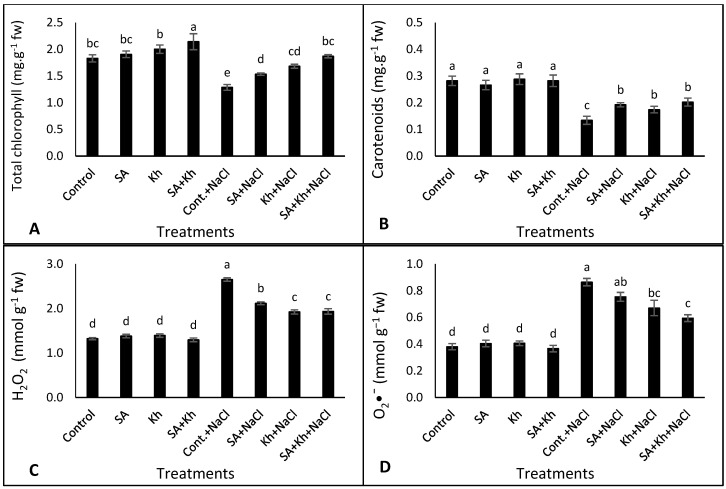
Effect of potassium humate (Kh), salicylic acid (SA), and their combination on (**A**) total chlorophyll, (**B**) carotenoids, (**C**) H_2_O_2_, and O_2_^•−^ (**D**) of common bean plants irrigated with 0 and 50 mM NaCl. Different letters indicate significant differences according to Duncan’s test (*p* < 0.05). Data are presented as means ± SE; *n* = 5.

Salinity stress reduced the content of total chlorophyll and carotenoids in common bean leaves under all treatments (Figure 3A,B). Moreover, the total chlorophyll content was significantly increased by only Kh + SA treatment under non-saline conditions compared to the control treatment. However, under saline stress, SA, Kh, and Kh + SA applications improved total chlorophyll content by 19.28%, 30.79%, and 45.41%, respectively, compared to the control. None of the treatments changed carotenoid content under the non-saline condition (Figure 3B). However, all treatments improved carotenoid content without significant differences compared to the control treatment. 

As shown in Figure 3C,D, H_2_O_2_ and O_2_^•−^ levels were increased by salinity stress under all treatments. Under the non-saline condition, neither H_2_O_2_ nor O_2_^•−^ levels were significantly different between any of the treatments. The accumulation of H_2_O_2_ in the leaves of common bean plants that were treated with Kh, SA, and Kh + SA was reduced by 20.37, 27.45, and 27.16%, respectively, compared to control treatment under NaCl stress. The same trend of results was recorded for O_2_^•−^ content. 

### 3.3. Effect of Kh and SA on the Activity of Antiaxidant Enzymes and Proline 

**Figure 4:**
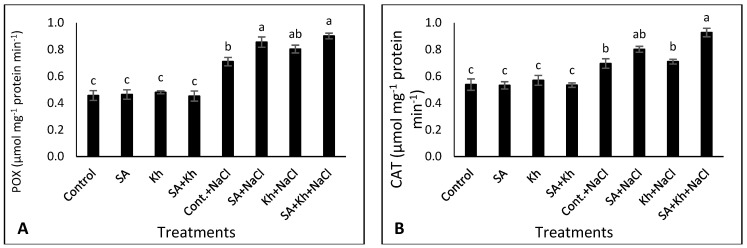

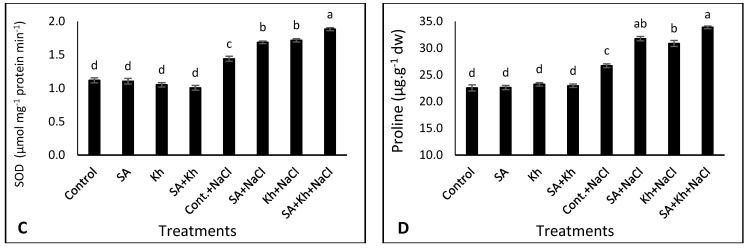
Effect of potassium humate (Kh), salicylic acid (SA), and their combination on (**A**) POX, (**B**) CAT, (**C**) SOD, and (**D**) proline content of common bean plants irrigated with 0 and 50 mM NaCl. Different letters indicate significant differences according to Duncan’s test (*p* < 0.05). Data are presented as means ± SE; *n* = 5.

As shown in Figure 4A–C, the activity of POX, CAT, and SOD enzymes in leaves was increased under saline stress compared to non-saline conditions for all treatments. Moreover, under non-saline conditions, there was no significant difference in the activity of any enzymes between treatments. However, under saline conditions, the POX activity in plants that were treated with Kh + SA was increased compared to the control. Similarly, SA, Kh, and Kh + SA treatments significantly increased the activities of CAT and SOD enzymes compared to the control under saline conditions. 

The proline concentration was increased under 50 mM NaCl stress compared with the non-saline condition in all treatments (Figure 3D). Under the non-saline condition, there was no significant difference in proline content between any of the treatments. However, all applications resulted in higher proline content values than the control treatment under saline conditions. 

### 3.4. Effect of Kh and SA on the GSH, AA, Na, and K 

**Figure 5:**
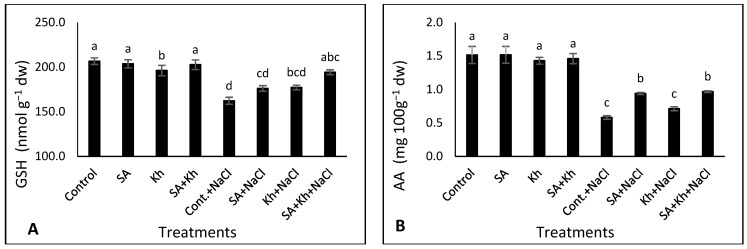

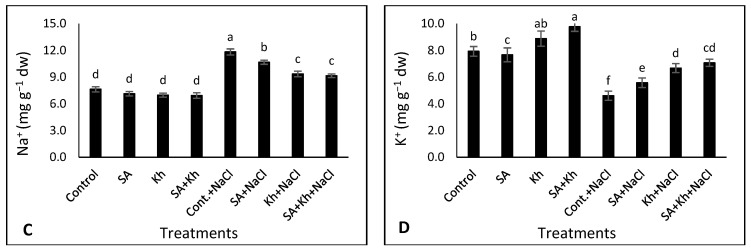
Effect of potassium humate (Kh), salicylic acid (SA), and their combination on (**A**) GSH, (**B**) AA, (**C**) Na, and (**D**) K content of common bean plants irrigated with 0 and 50 mM NaCl. Different letters indicate significant differences according to Duncan’s test (*p* < 0.05). Data are presented as means ± SE; *n* = 5.

The results presented in Figure 5A,B show that both GSH and AA in common bean seedlings were decreased by salinity treatment compared to the control treatment (non-saline condition). However, the SA + Kh application significantly increased GSH content under saline conditions compared to untreated seedlings. Furthermore, both SA and SA + Kh applications increased AA content in bean seedlings compared to the control under saline stress. 

The content of Na in the leaves of the bean seedlings irrigated with 50 mM NaCl was higher than in plants irrigated with regular water (Figure 5C). There was no significant difference in Na content between any of the treatments under non-saline conditions. Additionally, all treatments significantly reduced Na content compared with the control under saline stress. Under non-saline conditions, SA + Kh application resulted in higher K content than the control, whereas SA treatment decreased the K content (Figure 5D). However, SA, Kh, and Kh + SA applications increased the K content compared to the control under saline conditions. 

### 3.5. Principal Component Analysis and Heatmap Clustering

**Figure 6:**
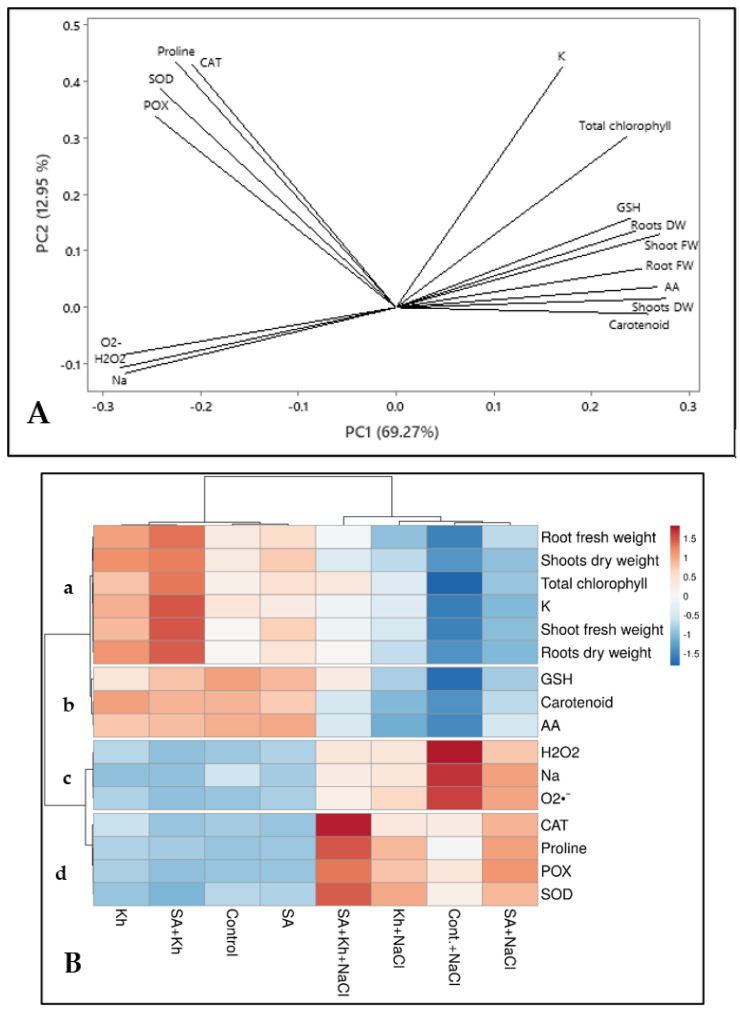
(**A**) Loading plot of all recorded parameters included in the PCA. (**B**) Heatmap analysis for treatments (columns) and tested parameters (rows). The numbers indicate the degree of colour. Blue refers to lower numerical values, and red refers to higher numerical values. The colour scale is located in the top-right corner of the chart.

A PCA biplot including PC1 and PC2 is presented in Figure 6A. The biplot visualizes how samples relate to each other, i.e., which samples are similar and which are different, as well as how each variable contributes to each principal component. PCA analysis accounted for 82% of the variance. PC1 and PC2 accounted for 69.27 and 12.95% of the total variability, respectively. Antioxidant enzymes and proline contributed significantly to PC2, and all vegetative growth and non-enzymatic antioxidants (GSH, AA, and carotenoids) showed inclinations toward PC1, including K and total chlorophyll. 

The heatmap shows that all growth and biochemical parameters under different treatments can be divided into four clusters (Figure 6B a, b, c, and d). Group a includes all vegetative growth parameters, total chlorophyll, and K. GSH, AA, and carotenoids are in group b, whereas H_2_O_2_, O_2_^•−^, and Na are in group c. Proline, CAT, POX, and SOD enzymes are categorized in group d. As shown in Figure 6B, group a indicates that vegetative parameters were reduced (dark blue) by salinity stress but enhanced by SA and Kh applications (light blue to red). GSH, AA, and carotenoid values (Figure 6B, group b) were reduced by salinity stress (blue) compared to non-saline conditions (red) and increased by SA and Kh applications under saline conditions. In contrast, H_2_O_2_, O_2_^•−^, and Na were increased by salinity stress (Figure 6B, group c). The double treatments (SA + Kh) showed the best results in reducing H_2_O_2_, O_2_^•−^, and Na contents under saline conditions (light blue values). Finally, the activity of antioxidant enzymes and proline increased under salinity stress compared to the non-saline condition (Figure 6B, group d). 

## 4. Discussion

### 4.1. Effect of Kh and SA on Growth

Salt stress reduces plant growth by affecting physiological processes such as photosynthesis, hormones, and enzyme activities [9]. Our study shows that salinity stress decreased the growth of common bean seedlings (Figure 2A–D). This reduction in growth might be due to several factors, such as ionic toxicity, osmotic pressure, limitation of elements absorption, a reduction in photosynthesis processes, and accumulation of Na in plant tissue [4,50]. In this study, SA, Kh, and Kh + SA applications increased plant growth parameters under normal and saline conditions. Recently, SA has been used in plant cultivation to mitigate the harmful effects of several abiotic stresses, such as salinity. This is due to the role of SA in enhancing the photosynthesis processes and mitigating cell membrane damage caused by salinity stress [51].

The application of SA might enhance the growth of seedlings by weakening the cell membrane. Furthermore, SA enhanced plant growth by maintaining the expected levels of indole acetic and gibberellic acid levels responsible for plant growth [52]. Additionally, previous studies indicated that SA application mitigates salinity and improves growth (plant fresh and dry weight) of some crops, such as kidney beans and cabbage [31,34]. 

In line with our results, previous works reported the positive role of Kh in enhancing plant growth of beans under normal and salinity stress [29,53,54]. The positive role of Kh in enhancing plant growth might be due to its role in increasing the organic matter of growth media, water availability, conserved mineral nutrients from leaching, and mineral absorption by plant roots [53,54,55]. Additionally, Kh was more effective than SA in improving plant growth (Figure 2). This could be due to the role of potassium in controlling many enzymes in plants [56], as well as the role of humate as a biostimulants [36]. Taken together, our results suggest that SA + Kh application was the most effective treatment. This result is in agreement with that reported by Shalaby et al. [36], who found that the combined application of SA + Kh significantly enhanced the plant growth of marigold plants. Moreover, heatmap analysis (Figure 6B, group a) showed that SA and Kh treatments increased vegetative growth parameters under saline conditions. 

### 4.2. Effect of Kh and SA on Leaf Pigments, O_2_^•−^, and H_2_O_2_

The decline in photosynthetic pigments is one of the most damaging effects of salt stress. Our results show that salinity stress reduced total chlorophyll and carotenoids (Figure 3A,B). Alzahrani et al. [57] confirmed our findings, reporting that saline conditions prevented the synthesis of chlorophyll and carotenoid in stressed plants. Salinity stress might also reduce chlorophyll fluorescence and cause a severe imbalance in stomatal function [58]. Additionally, salinity retards the absorption of magnesium, which is required for chlorophyll synthesis [59]. 

The results in this study (Figure 3A) and those of previous studies indicate that SA application enhances the chlorophyll content of kidney bean plants [31], tomatoes [60], cucumbers [61], and mungbean [62]. These results could be due to the role of SA in increasing the relative vitality of photosynthesis and decreasing initial fluorescence [63]. Furthermore, the role of Kh in enhancing chlorophyll and carotenoid contents under saline conditions was previously reported [29,64]. These findings could be due to the role of SA and Kh in enhancing photosynthesis biosynthesis (by protecting chloroplast pigments from salt toxicity through oxidative protection of chloroplasts) and improving the performance of enzymes during the chlorophyll process [51]. Bijanzadeh et al. [37] found that a combination treatment of SA and Kh improved chlorophyll and carotenoid content in corn plants, which supports our hypothesis that the combination of the two compounds is more effective than the individual compounds. Furthermore, heatmap analysis (Figure 6B) showed that SA and Kh treatments under saline conditions increased leaf pigments. 

Environmental stresses, especially salinity and drought, encourage the production of reactive oxygen species (ROS), including O_2_^•−^ and H_2_O_2_, which promote the tolerance of plants to these stresses [62]. Figure 3C,D show a clear trend of increasing ROS under salinity stress compared with the non-saline condition. A similar result was recorded by Nawaz et al. [62], who found that salinity increased ROS levels in mungbean shoots. However, in this study, the application of SA and Kh retarded the production of H_2_O_2_ under saline conditions. This result is in agreement with that reported by Nawaz et al. [62], who showed that SA application decreased the H_2_O_2_ content in mungbean shoots under saline conditions. SA may have played a vital role in ROS scavenging and reduced H_2_O_2_ content, which maintains plant growth under adverse conditions [65]. Moreover, Kh effectively reduced ROS generated by oxidative stresses in maize [66] and strawberries [38]. Additionally, PCA (Figure 6A) and heatmap analysis (Figure 6A, group c) showed that O_2_^•−^, H_2_O_2_, and Na are closely clustered in the same group.

### 4.3. Effect of Kh and SA on the Activity of Antioxidant Enzymes and Proline

Under abiotic stresses, several mechanisms of plants mitigate these stresses. One of these mechanisms is enhancement of the activity of antioxidant enzymes such as CAT, SOD, and POX, which play a vital role in scavenging ROS [67]. These findings are consistent with our results presented in Figure 4A–C, indicating an increase in the activity of antioxidant enzymes under saline conditions. Similar results were recorded in strawberry plants [33]. 

The results of this study indicate that the application of SA increased the activity of antioxidant enzymes under saline stress. Treatment with SA might stimulate the activity of antioxidant enzymes, reducing the harmful effects of ROS under saline stress [68]. Previous studies indicated that SA application increases the activity of antioxidant enzymes under biotic stress conditions [36,69,70,71]. We also recorded an increase in the activities of CAT, SOD, and POX as a result of Kh treatment (Figure 4A–C). These results match those observed in an earlier study by Hemida et al. [53], who found that Kh application increased the activities of CAT, SOD, and POX enzymes in common bean plants under salinity conditions. The increased activity of antioxidant enzymes with the increasing AA and GSH (Figure 5A,B) by SA + Kh application could encourage the normal growth of plants under environmental stresses. Potassium also plays a vital role in plant growth and regulates many physiological processes by controlling their enzymes [72], which could explain the effective role of Kh in enhancing antioxidant enzyme activity [36]. 

It is well known that proline accumulates in plant tissue under unfavourable environmental conditions, especially salinity and drought [73]. Proline protects proteins, membranes, and cellular structures from damage by scavenging reactive oxygen species (ROS) in tissue [74,75,76]. Many previous works recorded an increase in proline content in some crops, such as tomatoes [4], broad bean [9], and green beans [29], under saline conditions. Similarly, our results presented in Figure 3C and Figure 5 show that salinity increased proline content in common bean leaves. The increase in proline content by SA application in mungbean was previously recorded [62]. As a growth regulator, SA could help regulate osmotic pressure, protect cell membranes from damage, and scavenge ROS under stress conditions [62]. Additionally, previous work indicated that Kh application increased proline content in pea leaves under saline stress [55]. In this study, Kh improved the activity of antioxidant enzymes and proline content, which could mitigate salinity [25]. Moreover, PCA (Figure 6A) and heatmap analysis (Figure 6B, group d) showed that the activity of antioxidant enzymes and proline are in the same group that increased by SA and Kh treatments under saline conditions compared to the control plants.

### 4.4. Effect of Kh and SA on the GSH, AA, Na^+^, and K^+^

In this study, GSH and AA were decreased by salinity but increased by Kh and SA application (Figure 5A,B). It has been reported that AA and GSH protect cell membranes from oxidative damage by reacting with O_2_^•−^ and H_2_O_2_ [77,78]. The application of Kh mitigates the adverse effects of salinity by improving the absorption of elements, enhancing plant growth [55], and motivating the plant defence system against stresses [53]. Previous studies indicated that Kh application increased antioxidant activity in pepper plants under saline conditions [79]. The role of SA treatment in salinity resistance was reported in some previous studies [33,34,35]. Increasing GSH and AA contents in plants by SA application could enhance a mechanism to protect plants from the accumulation of ROS under stress conditions [80]. 

Salinity affects the absorption and accumulation of elements in plant tissue [10]. It is well known that uptakes of nutrients (N, P, K^+^, Ca, and Mg, as well as microelements) in the rhizosphere area is negatively affected by high levels of Na and Cl [4]. In this study, salinity increased the level of Na^+^ and decreased the K^+^ content in common bean leaves (Figure 4C,D). Saidimoradi et al. [38] obtained a similar pattern of results, finding that salinity stress reduced the uptake of K^+^ by strawberry plants. Maintaining adequate K^+^ levels in plants could mitigate adverse salinity conditions [11,81,82]. Under the saline condition, our results show that Na^+^ content in leaves was decreased, whereas Kh and SA treatments and their combination increased K^+^ content (Figure 4C,D). This result is in line with previous studies by Gunes et al. [83] and Roshdy et al. [33], who found that SA application reduced Na^+^ levels and increased K levels in maize and strawberry plants, respectively, under saline conditions. Additionally, soil Kh application improved the absorption of K^+^ and decreased the uptake of Na^+^ in shoots of strawberry plants Saidimoradi et al. [38]. Kh also contains K^+,^ which is known to be responsible for salinity resistance, owing to its competition with sodium in terms of binding and maintaining plant water status [84]. The adsorption of Na by humic compounds as a result of Kh application helps to reduce the content of Na in common bean shoots and allows more K^+^ to be absorbed by the roots [85].

### 4.5. Effect of Combined Application (Kh + SA) 

The results of this study show that there are no significant differences between SA + Kh and Kh applications in shoot fresh weight, shoot dry weight, root dry weight, total chlorophyll, total carotenoids, H_2_O_2_, O_2_^•−^, POX, GSH, Na^+^, or K^+^ under saline conditions. This might be due to the role of K^+^ and humate in both treatments in improving plant growth. However, with respect to some other parameters (fresh weight, CAT, proline, AA, and SOD) SA + Kh treatment was more effective than the individual applications. Thus, our results suggest that soil application of Kh plus SA foliar application can mitigate salinity stress of bean seedlings. More studies are required to prove the effectiveness of the combined application of SA and Kh in enhancing salinity tolerance in bean plants. 

## 5. Conclusions

**Figure 7:**
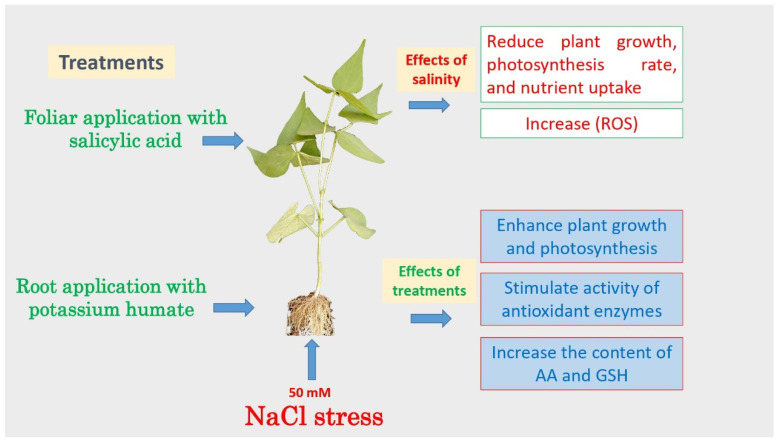
Simplified chart illustrating the effect of Kh and SA applications on the growth and chemical changes of common bean seedlings grown under salinity stress.

Oxidative damage resulting from salinity stress was observed, as evidenced by a reduction in shoot and root growth and pigments such as chlorophyll and carotenoids (Figure 7). However, the application of SA, Kh, and SA + Kh enhanced the resistance of common bean to salinity stress by reducing Na, O_2_^•−^, and H_2_O_2_ contents in shoots and increasing content, the activity of the antioxidant enzymes, AA, GSH, and proline content. The most effective treatment was the combined treatment. Thus, combining SA + Kh can effectively mitigate salinity stress in common bean plants.

## Data Availability

All data are available within the manuscript.
